# Melatonin supplementation protects against traumatic colon injury by regulating SERPINA3N protein expression

**DOI:** 10.1002/imt2.141

**Published:** 2023-10-24

**Authors:** Bo Cao, Jing‐Wang Gao, Qing‐Peng Zhang, Xing‐Ming Xu, Rui‐Yang Zhao, Hang‐Hang Li, Bo Wei

**Affiliations:** ^1^ Department of General Surgery, First Medical Center Chinese PLA General Hospital Beijing China; ^2^ Medical School of Chinese PLA Beijing China

**Keywords:** gut homeostasis, melatonin, microbiota dysbiosis, posttraumatic survival time, SERPINA3N, traumatic colon injury

## Abstract

Traumatic colon injury (TCI) is a typical injury with high mortality. Prolongation of the intervention time window is a potentially useful approach to improving the outcomes of TCI casualties. This study aimed to identify the pathological mechanisms of TCI and to develop effective strategies to extend the survival time. A semicircular incision was made to prepare a TCI model using C57BL/6 mice. An overview of microbiota dysregulation was achieved by metagenome sequencing. Protein expression reprogramming in the intestinal epithelium was investigated using proteomics profiling. The mice that were subjected to TCI died within a short period of time when not treated. Gut symbiosis showed abrupt turbulence, and specific pathogenic bacteria rapidly proliferated. The protein expression in the intestinal epithelium was also reprogrammed. Among the differentially expressed proteins, SERPINA3N was overexpressed after TCI modeling. Deletion of *Serpina3n* prolonged the posttraumatic survival time of mice with TCI by improving gut homeostasis in vivo. To promote the translational application of this research, the effects of melatonin (MLT), an oral inhibitor of the SERPINA3N protein, were further investigated. MLT effectively downregulated SERPINA3N expression and mitigated TCI‐induced death by suppressing the NF‐κB signaling pathway. Our findings prove that preventive administration of MLT serves as an effective regimen to prolong the posttraumatic survival time by restoring gut homeostasis perturbed by TCI. It may become a novel strategy for improving the prognosis of patients suffering from TCI.

## Highlights

- SERPINA3N expression is significantly upregulated in the intestinal epithelium of traumatic colon injury (TCI) mice.
- Restoration of ectopic SERPINA3N expression prolongs the survival time by attenuating the dysregulation of gut homeostasis in TCI mice.
- Preventive administration of melatonin, as an oral inhibitor of SERPINA3N expression, is a potentially useful approach to treat patients with TCI.

## Graphical abstract

Traumatic colon injury leads to significant disruption of gut homeostasis within a short period of time, indicated by inflammatory responses, intestinal barrier hyperpermeability, and microbiota dysregulation. Melatonin administration can restore gut homeostasis by suppressing SERPINA3N expression in the intestinal epithelium, subsequently prolonging the survival time of mice with traumatic colon injury.

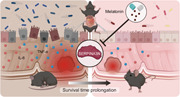

## INTRODUCTION

Traumatic colon injury (TCI) is a type of abdominal trauma that poses a major threat to human health. Due to the nature of warfare, the incidence of TCI has dramatically increased in disaster‐hit areas and battlefields, and for a large number of patients, TCI leads to poor prognosis and quality of life [1, 2, 3]. Compared with the convenience of casualty hospitalization during peacetime, disaster‐hit and battlefield areas are complicated and unpredictable. The evacuation speeds and stability cannot be guaranteed. TCI patients may die or show rapid disease progression within a short period of time [4, 5]. Current advances in first‐aid equipment and skills have reached an impasse, which hinders the progress of military medical services [6]. Therefore, prolonging posttraumatic survival time and buying time for evacuation to station hospitals could serve as effective approaches to increase the survival rates of TCI patients. Considering the organizational discipline of the army and the foreseeability of warfare, the development of preventive medical regimens may be a promising approach. However, research on this scientific issue is still lacking.

Melatonin (MLT) is the main neuroendocrine product of the pineal gland, synthesized from tryptophan and predominantly secreted during the night. This drug plays an important role in regulating circadian rhythms. The administration of exogenous MLT has been used for the adjuvant treatment of insomnia [7]. With the development of relevant research, accumulating evidence has revealed its other properties, including antioxidation, induction of cancer apoptosis, immunomodulation, and alleviation of senescence [8, 9, 10, 11]. Importantly, MLT has shown the potential to restore intestinal functions. For instance, MLT can ameliorate sepsis‐induced intestinal injury through inhibition of oxidative stress, mitochondrial‐function protection, and autophagic induction [12]. The progression of necrotizing enterocolitis is also alleviated by MLT by activation of the AMPK/SIRT1 signaling pathway and prevention of Th17/Treg imbalance [13]. Hence, MLT may serve as an effective drug to, at least partly, restore gut homeostasis when the intestinal barrier is damaged.

SERPINA3N, also referred to as α‐1‐antichymotrypsin, is a member of the serpin family of protease inhibitors [14]. SERPINA3N is highly expressed in multiple organs, such as the brain, lung, muscle, and kidney [15]. The pathophysiological functions of SERPINA3N protein have been widely investigated. For instance, dysregulation of SERPINA3N expression is closely associated with neuropathological disorders [16, 17]. The overexpression of SERPINA3N has been identified in various types of cancers and may become a biomarker for cancer diagnosis [18]. Moreover, SERPINA3N upregulation contributes to the progression of dilated myocarditis in response to inflammatory responses [19]. However, the role of SERPINA3N during the response to TCI is not well characterized.

In this study, microbiome and proteomics analyses were jointly conducted to explore the mechanisms underlying TCI‐induced death based on a TCI mouse model. A series of in vivo experiments were further performed to elucidate the critical functions of SERPINA3N in the intestinal epithelium during the rapid progression of TCI. The efficacy of MLT, as an effective inhibitor of SERPINA3N, was examined to facilitate the translational application of this research. This preventive medical regimen may provide a novel strategy for managing TCI casualties during disaster rescue and military operations.

## RESULTS

### The abundance of pathogenic bacteria is abruptly increased after TCI modeling

**Figure 1:**
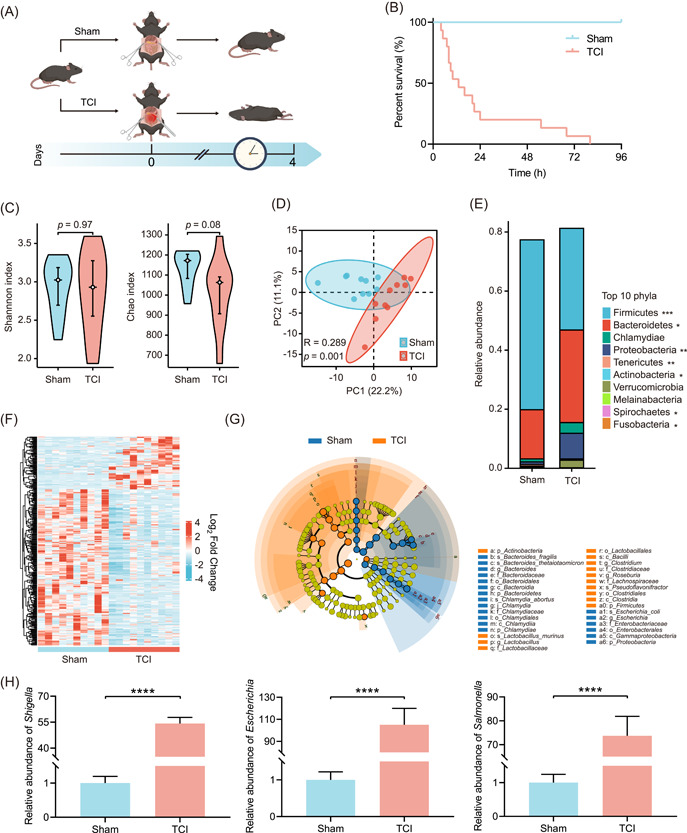
The abundance of pathogenic bacteria is abruptly increased after TCI modeling. (A) Flow diagram used to establish sham and TCI mouse models. TCI was induced in mice by the incision of the colon that was 3 cm from the cecum end. Sham mice were only subjected to laparotomy without colon incision. (B) Survival curve showing the survival differences between sham and TCI groups (*n* = 15 per group). (C) Violin plots comparing alpha diversities of gut microbiota in the sham and TCI groups. (D) Scatter plot comparing the beta diversity of gut microbiota as in (C). (E) Stacked histogram showing the relative abundances of top 10 phyla of gut microbiota as in (C). (F) Heatmap showing the bacterial genera with different abundances as in (C) (*p* < 0.05). (G) Cladogram showing the differential bacteria as in (C). The circles from the inside to the outside indicate the taxonomic ranks from phylum to genus. The blue and orange points indicate the bacteria with important functions in the corresponding groups. The yellow points indicate the species without significant differences in relative abundances. (H) qRT‐PCR analysis to determine the relative abundances of *Shigella*, *Escherichia*, and *Salmonella* in intestinal contents of sham and TCI mice (*n* = 15 per group). *p < 0.05, **p < 0.01, ***p < 0.001, ****p < 0.0001. qRT‐PCR, quantitative real‐time polymerase chain reaction; TCI, traumatic colon injury.

To gain insights into the effects of TCI on intestinal microbiota, we first induced TCI in mice and performed survival follow‐up (Figure 1A). The mortality rate of TCI mice reached 100% within 96 h, while there were no deaths in the sham group. These data demonstrate that TCI can be a lethal injury for mice without medical intervention (Figure 1B). Next, to investigate microbiome alterations induced by TCI, we set up another batch of experimental groups, including sham and TCI mice, and samples were collected after 24 h of modeling. Metagenome profiling was conducted to compare differences in gut microbiota compositions between the sham and TCI groups. There were no significant differences in alpha diversity, as shown by the Shannon and Chao indices (Figure 1C). Nevertheless, the principal coordinates analysis indicated an obvious separation in the intestinal microbiota structure between these two groups (Figure 1D). These results prove that the damage to colonic integrity induces a rapid shift in dominant bacteria, whereas species evenness and the amounts are unchanged in the short term.

We next analyzed the alterations in specific bacteria induced by TCI. The histogram shows the compositions of the bacterial communities of the sham and TCI mice (Figure 1E). The heatmap showed that the abundances of more than 300 genera showed appreciable changes (Figure 1F). Moreover, linear discriminant analysis effect size (LEfSe) analysis was carried out to identify operational taxonomic units (OTUs) and taxa with different abundances in sham and TCI mice. The dominant bacteria at different levels were affected by TCI (Figure 1G). We found that the abundances of three typically pathogenic bacteria, *Shigella*, *Escherichia*, and *Salmonella*, abruptly increased in the intestines of TCI mice. Quantitative real‐time polymerase chain reaction (qRT‐PCR) analysis was performed to confirm these results as shown by metagenome profiling. The abundances of *Shigella*, *Escherichia*, and *Salmonella* increased by 54.194 ± 3.581, 105.067 ± 14.860, and 73.763 ± 8.102 fold, respectively (Figure 1H). Moreover, the abundances of two kinds of opportunistic pathogens, *Cronobacter* and *Proteus*, were also upregulated according to the high‐throughput data, which was then confirmed by qRT‐PCR analysis (*Cronobacter*: 29.914 ± 1.894; *Proteus*: 14.847 ± 1.122, Supporting Information: Figure S1). These data collectively prove that TCI can disrupt gut microbiota symbiosis and that pathogenic bacteria rapidly proliferate during this pathological process.

### TCI reprograms the protein expression profile of the intestinal epithelium following TCI challenge

**Figure 2:**
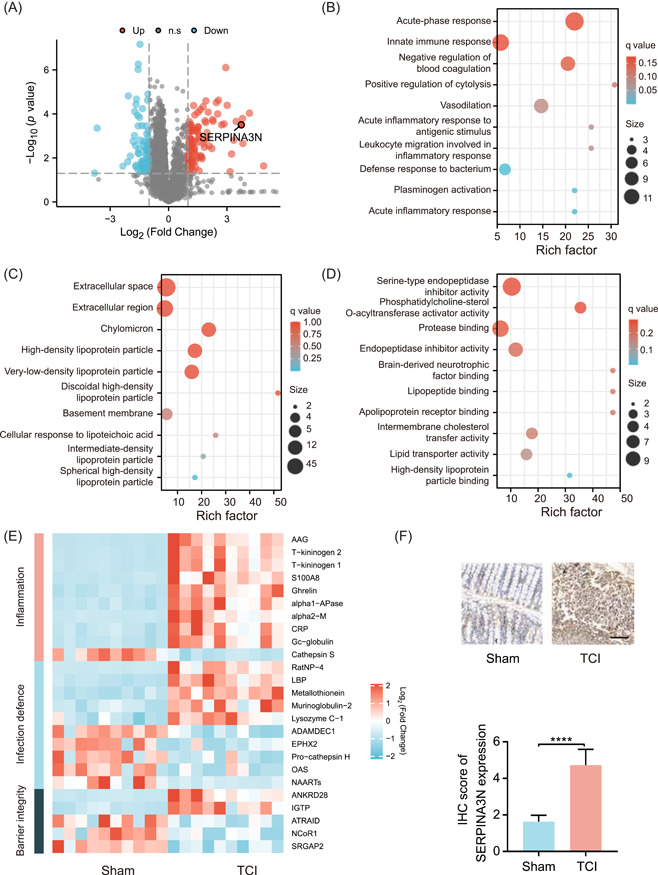
TCI reprograms the protein expression profile of the intestinal epithelium following TCI challenge. (A) Volcano plot showing the differential proteins in intestinal epithelium (*n* = 10 per group, log_2_FC < −1 or >1, *p* < 0.05). (B–D) Bubble plots showing the top enriched 10 items of BP, CC, and MF categories by GO analysis. (E) Heatmap showing differential proteins reported to be associated with inflammation, infection defense, and barrier integrity. (F) IHC examination used to detect the SERPINA3N expression in the intestinal epithelium of sham and TCI groups. The histogram is displayed below the IHC pictures. Scale bar: 100 μm. ****p < 0.0001. BP, biological process; CC, cellular component; GO, Gene Ontology; IHC, immunohistochemistry; MF, molecular functional; TCI, traumatic colon injury.

The TCI‐induced alterations in the protein profile of the intestinal epithelium were not previously investigated. Herein, we used proteomics profiling to reveal the mechanisms underlying protein expression reprogramming, followed by TCI modeling. The results showed 71 upregulated proteins and 40 downregulated proteins in the epithelium of TCI mice (log_2_FC < −1 or >1, *p* < 0.05). Among all the significantly altered proteins, SERPINA3N was found to be upregulated by over 10‐fold in the intestinal epithelium (Figure 2A). Furthermore, these differentially expressed proteins were found to be enriched by Gene Ontology (GO) analysis, and can be classified into biological process (BP), cellular component (CC), and molecular function (MF) categories. According to the top 10 enriched categories in BP analysis, differentially expressed proteins detected among the sham and TCI groups were primarily involved in inflammatory responses (Figure 2B). Extracellular processes and lipid metabolism were the representative terms in CC (Figure 2C). As shown by the MF analysis, protein modification and catabolism were mostly enriched, and the impact of TCI on lipid metabolism was also confirmed (Figure 2D). To specifically identify the pathological processes during TCI progression, we reviewed the literature on the functions of the differentially expressed proteins. Most of them could be clustered into three aspects: inflammation, infection defense, and barrier integrity. These related proteins are shown in the heatmap (Figure 2E). IHC examination was then performed to validate the upregulated expression of SERPINA3N in the intestinal epithelium (Figure 2F).

### Knockout of *Serpina3n* protein prolongs the survival time of TCI mice

**Figure 3:**
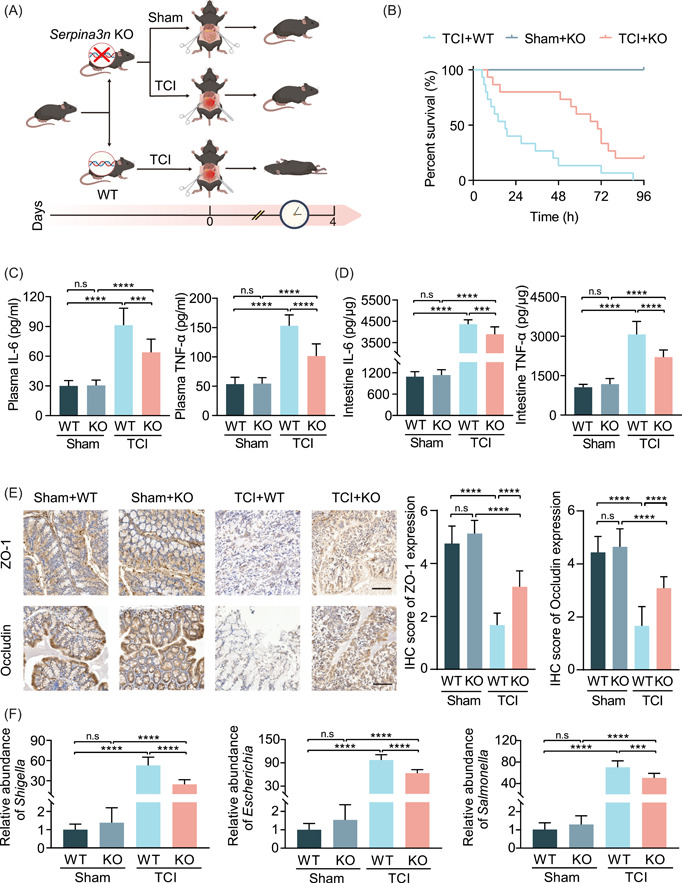
Knockout of SERPINA3N protein prolongs the survival time and gut homeostasis of TCI mice. (A) Flow diagram used to establish the *Serpina3n* KO mice that were subjected to sham or TCI operation and WT mice that were subjected to the TCI operation (*n* = 15 per group). (B) Survival curve comparing the survival time of mice as in (A). (C) ELISA assay used to detect the concentrations of IL‐6 and TNF‐α in the plasma of WT or *Serpina3n* KO mice that were subjected to sham or TCI operation (*n* = 15, 15, 11, and 13, respectively). (D) ELISA assay used to detect the concentrations of IL‐6 and TNF‐α in the intestines of mice as in (C). (E) IHC examination used to determine ZO‐1 and occludin expression in the intestinal epithelium of mice as in (C). The histogram is displayed on the right of the pictures. Scale bar: 100 μm. (F) qRT‐PCR analysis used to determine the relative abundances of *Shigella*, *Escherichia*, and *Salmonella* in the intestinal contents of mice as in (C). ***p < 0.001, ****p < 0.0001. ELISA, enzyme‐linked immunosorbent assay; IHC, immunohistochemistry; IL‐6, interleukin‐6; KO, knockout; n.s, not significant; qRT‐PCR, quantitative real‐time polymerase chain reaction; TCI, traumatic colon injury; TNF‐α, tumor necrosis factor‐α; WT, wild type.

Regarding the ectopic expression of SERPINA3N during TCI progression, it was speculated that restoration of a low level of SERPINA3N expression might alleviate TCI aggravation and prolong posttraumatic survival time. We first established Caco‐2 cells with stable knockdown and overexpression of *Serpina3n* for in vitro experiments. The interfering effects were verified by WB analysis (Supporting Information: Figure S2A). Transwell assays showed that downregulation of *Serpina3n* expression enhanced the migration of Caco‐2 cells and that *Serpina3n* overexpression led to significant suppression of cell migration (Supporting Information: Figure S2B,C). To further detect its regulatory functions in vivo, we generated *Serpina3n* KO mice and conducted IHC to confirm the loss of SERPINA3N in the KO mice (Supporting Information: Figure S3). TCI modeling was again performed using wild‐type (WT) and *Serpina3n* KO mice (Figure 3A). Posttraumatic survival analysis showed that *Serpina3n* deletion contributed to survival prolongation in TCI mice (TCI+*Serpina3n* KO versus TCI+WT: hazard ratio [HR] = 0.361, 95% confidence interval [CI] 0.160–0.815, *p* = 0.004) (Figure 3B). However, SERPINA3N is expressed in multiple organs. To further explore the regulatory role of SERPINA3N in the gut, we generated conditional knockout (CKO) mice with the absence of *Serpina3n* expression in the gut epithelium (Supporting Information: Figure S4A). The TCI model was subsequently established using *Serpina3n* CKO, *Serpina3n* KO, and control mice. The posttraumatic survival time of *Serpina3n* CKO mice was significantly extended compared to the control mice (TCI+*Serpina3n* CKO versus TCI: HR = 0.325, 95% CI 0.139–0.756, *p* = 0.009). There were no statistical differences between the survival time of *Serpina3n* CKO mice and *Serpina3n* KO mice (TCI+*Serpina3n* CKO versus TCI+*Serpina3n* KO: HR = 1.186, 95% CI 0.520–2.704, *p* = 0.686) (Figure S4B). These results suggest that deletion of *Serpina3n* in the gut alleviated TCI progression, rather than knockout of *Serpina3n* in other organs. Taken together, both in vitro and in vivo experiments indicate that abrupt upregulation of *Serpina3n* plays an important role in TCI progression toward death. Elimination of *Serpina3n* efficaciously extended the posttraumatic survival time of TCI mice.

### Ablation of *Serpina3n* restores gut homeostasis perturbed by TCI

Since there are close associations between gut homeostasis and the survival time of TCI mice, as indicated by proteomics profiling, we then investigated the effects of *Serpina3n* knockout on inflammation, intestinal hyperpermeability, and bacterial dysregulation. Another batch of experimental groups was established and sample collection was conducted after 24 h of modeling. ELISA assay was first used to determine the concentrations of tumor necrosis factor‐α (TNF‐α) and interleukin‐6 (IL‐6) in the WT and *Serpina3n* KO mice that were subjected to sham or TCI modeling. The results showed that TCI induced a marked increase in TNF‐α and IL‐6 in both plasma and intestines. Deletion of *Serpina3n* had no significant impacts on these indicators in sham mice, whereas it partly reversed the increase of TNF‐α and IL‐6 induced by TCI (Figure 3C,D). Moreover, C‐reactive Protein (CRP) is an early indicator of inflammatory conditions. TCI led to a significant increase of plasma CRP, and *Serpina3n* deletion partly mitigated the effects of TCI on CRP levels (Supporting Information: Figure S5A).

Plasma diamine oxidase (DAO), D‐lactate, and endotoxin were selected as markers of gut barrier permeability. TCI modeling resulted in drastic increases in the three indicators, which were partly inhibited by knockout of *Serpina3n* in TCI mice (Supporting Information: Figure S5B). ZO‐1 and occludin are tight junction proteins that are widely expressed in the intestinal barrier. IHC showed that TCI reduced ZO‐1 and occludin expression in the epithelium. The expression levels of these molecules were found to be partly restored in *Serpina3n* KO mice that were subjected to TCI (Figure 3E).

To investigate the effects of *Serpina3n* on microbiota dysbiosis, we chose five pathogenic or opportunistic pathogenic genera, *Shigella*, *Escherichia*, *Salmonella*, *Cronobacter*, and *Proteus*, as representative markers and detected these using qRT‐PCR. Similar to the results of inflammation and barrier permeability, elimination of *Serpina3n* partly suppressed their proliferation in the intestines (TCI+WT vs. TCI+KO: *Shigella*: 52.765 ± 12.250 vs. 24.541 ± 6.97; *Escherichia*: 97.493 ± 13.401 vs. 63.588 ± 8.990; *Salmonella*: 70.256 ± 12.068 vs. 50.665 ± 8.398; *Cronobacter*: 30.703 ± 2.237 vs. 16.436 ± 2.264; *Proteus*: 14.797 ± 1.357 vs. 8.368 ± 0.850. Figures 3F and Supporting Information: Figure S5C). These data collectively demonstrate that deletion of *Serpina3n* alleviates the dysregulation of gut homeostasis in vivo, characterized by inflammatory attenuation, barrier integrity improvement, and dysbiosis inhibition.

### Administration of MLT extends the survival time of TCI mice by downregulation of SERPINA3N expression

**Figure 4:**
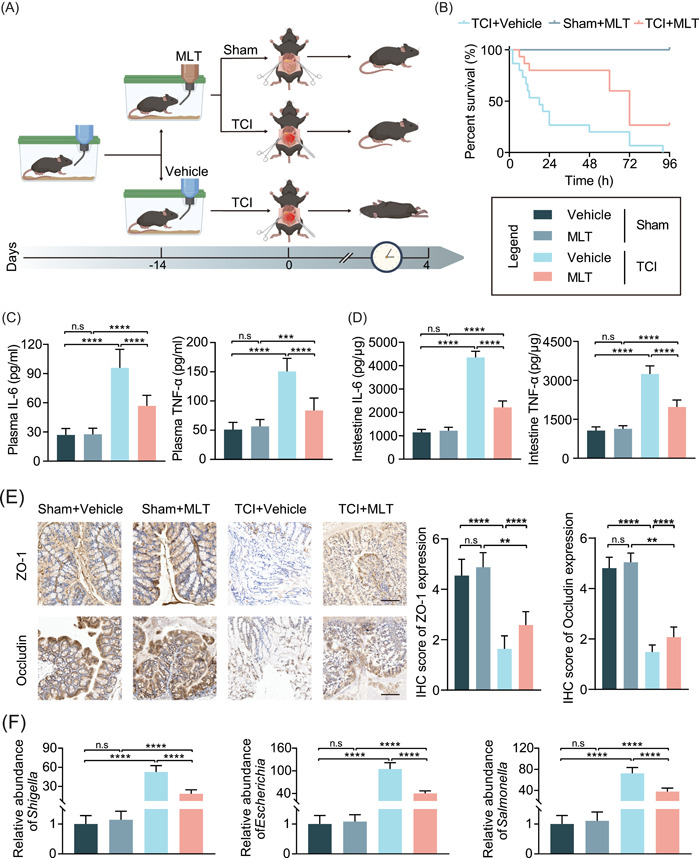
Administration of MLT extends the survival time and gut homeostasis of TCI mice by downregulation of SERPINA3N expression. (A) Flow diagram used to generate the mice in the sham or TCI groups that were administered with vehicle or MLT as indicated (*n* = 15 per group). (B) Survival curve comparing the survival time of mice as in (A). (C) ELISA assay used to detect the concentrations of IL‐6 and TNF‐α in the plasma of mice in the sham or TCI group that were administered with vehicle or MLT (*n* = 15, 15, 10, and 13, respectively). (D) ELISA assay used to detect the concentrations of IL‐6 and TNF‐α in the intestines of mice as in (C). (E) IHC examination used to determine ZO‐1 and occludin expression in the intestinal epithelium of mice as in (C). The histogram is displayed on the right of the pictures. Scale bar: 100 μm. (F) qRT‐PCR analysis used to determine the relative abundances of *Shigella*, *Escherichia*, and *Salmonella* in intestinal contents of mice as in (C). **p < 0.01, ***p < 0.001, ****p < 0.0001. ELISA, enzyme‐linked immunosorbent assay; IHC, immunohistochemistry; IL‐6, interleukin‐6; MLT, melatonin; n.s, not significant; qRT‐PCR, quantitative real‐time polymerase chain reaction; TCI, traumatic colon injury; TNF‐α, tumor necrosis factor‐α; WT, wild type.

MLT has been reported to suppress SERPINA3N expression in vivo [20]. Based on this evidence, we speculated that administration of MLT could prolong the survival time of TCI mice. SERPINA3N expression was measured using WB analysis in Caco‐2 cells pretreated with gradient doses of MLT. With increasing MLT concentrations, SERPINA3N protein levels gradually decreased (Supporting Information: Figure S6A). The migration capabilities of Caco‐2 cells were subsequently inhibited by MLT treatment in a significant dose‐dependent manner (Supporting Information: Figure S6B). Next, in vivo, the mice were orally administered with MLT for 14 days before TCI modeling (Figure 4A). The mice proactively treated with MLT showed better survival time than the TCI group (TCI+MLT vs. TCI+vehicle: HR = 0.374, 95% CI 0.166‐0.842, *p* = 0.0039, Figure 4B). The suppressive effects of MLT on SERPINA3N expression were confirmed using IHC examinations (Supporting Information: Figure S6C).

Furthermore, to confirm that SERPINA3N is a downstream target of MLT, Caco‐2 cells with stable overexpression of SERPINA3N were created and treated with MLT. WB analysis showed that MLT effectively mitigated SERPINA3N expression in both control Caco‐2 cells and Caco‐2 cells overexpressing SERPINA3N (Supporting Information: Figure S7A). For the migratory capability, MLT could also suppress the inhibitory effects of upregulation of SERPINA3N expression (Supporting Information: Figure S7B,C). In vivo, survival analysis demonstrated that MLT and *Serpina3n* KO prolonged the survival time of TCI mice, which was consistent with previous results. However, no significant survival benefits from MLT were observed in TCI mice with the deletion of *Serpina3n* (Supporting Information: Figure S7D). The data collectively prove that MLT extends the posttraumatic survival time of TCI mice by targeting SERPINA3N expression.

### MLT protects against TCI by restoring the disruption of gut homeostasis

Because of the associations between SERPINA3N and gut homeostasis, we conducted a series of experiments to detect indicators of inflammation, intestinal permeability, and pathogenic bacteria for the determination of homeostasis status. MLT reduced TNF‐α, IL‐6, and CRP, suggesting that the systemic and gut inflammatory responses were effectively inhibited (Figure 4C,D and Supporting Information: Figure S8A). The levels of three barrier permeability indicators, DAO, D‐lactate, and endotoxin, were downregulated (Supporting Information: Figure S8B). ZO‐1 and occludin protein expression was also rescued by MLT administration (Figure 4E). In the examinations of pathogenic bacterial abundances, a significant delay in *Shigella*, *Escherichia*, *Salmonella*, *Cronobacter*, and *Proteus* proliferation was observed (TCI+vehicle vs. TCI+MLT: *Shigella*: 52.705 ± 9.904 vs. 18.503 ± 6.214; *Escherichia*: 104.730 ± 17.057 vs. 40.217 ± 6.607; *Salmonella*: 72.386 ± 11.031 vs. 37.927 ± 6.503; *Cronobacter*: 31.253 ± 3.602 vs. 15.594 ± 2.350; and *Proteus*: 15.514 ± 1.702 vs. 7.97 ± 1.101, Figure 4F and Supporting Information: Figure S8C).

### MLT suppresses SERPINA3N expression by inhibiting the NF‐κB signaling pathway

**Figure 5:**
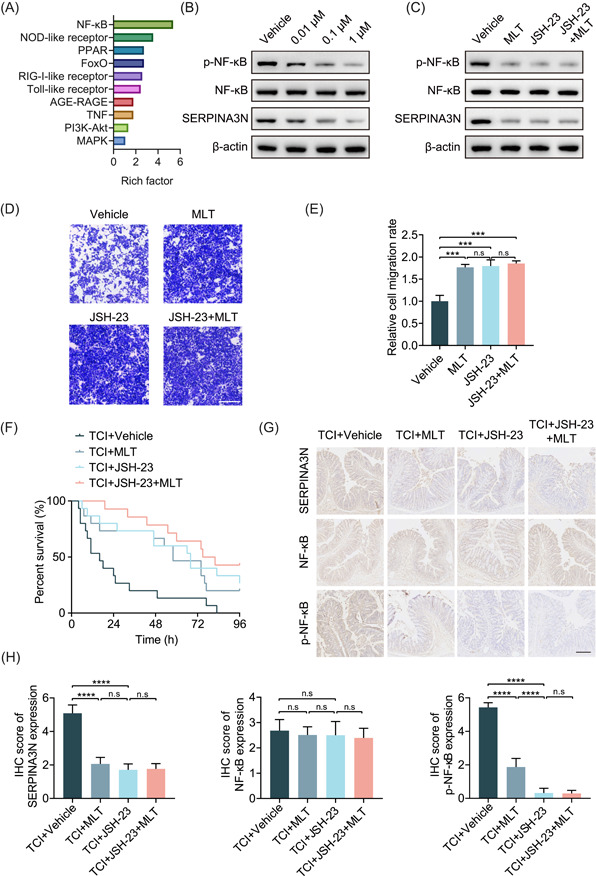
MLT suppresses SERPINA3N expression by inhibiting the NF‐κB signaling pathway. (A) Histogram showing the signaling pathways that ranked top 10 according to KEGG analysis based on proteomics profiling. (B) WB analysis used to determine the p‐NF‐κB, NF‐κB, and SERPINA3N expression in Caco‐2 cells treated with 0.01, 0.1, and 1 μM MLT for 24 h. (C) WB analysis used to determine the p‐NF‐κB, NF‐κB, and SERPINA3N expression in Caco‐2 cells treated with vehicle, MLT, 100 μM JSH‐23, or MLT plus 100 μM JSH‐23. (D and E) Transwell assay used to determine cell migration capabilities of cells as in (C). The histogram is displayed on the right. Scale bar: 100 μm. (F) Survival curve comparing the survival time of TCI mice treated with vehicle, MLT, 3 mg/kg JSH‐23, and MLT plus 3 mg/kg JSH‐23 (*n* = 15 per group). (G) IHC examination used to determine SERPINA3N, p‐NF‐κB, and NF‐κB expression in the intestinal epithelium of mice that were subjected to the same treatment as in (F). Scale bar: 100 μm. (H) Histogram showing IHC scores as in (G). ***p < 0.001, ****p < 0.0001. KEGG, Kyoto Encyclopedia of Genes and Genomes; IHC, immunohistochemistry; MLT, melatonin; n.s not significant; TCI, traumatic colon injury; WB, western blot.

To investigate the mechanisms underlying MLT‐downregulated SERPINA3N expression in the gut epithelium of TCI mice, we conducted Kyoto Encyclopedia of Genes and Genomes (KEGG) analysis to clarify alterations in the signaling pathways of gut epithelium. The results indicated that the NF‐κB signaling pathway of the gut epithelium ranked first among the altered pathways after TCI modeling (Figure 5A). Thus, we speculated that MLT might prolong the survival time of TCI mice by reducing activation of the NF‐κB signaling pathway. To validate this assumption, we first examined the activity of the NF‐κB signaling pathway in Caco‐2 cells treated with different doses of MLT. The phosphorylation of NF‐κB decreased with increasing MLT doses (Figure 5B).

**Figure 6:**
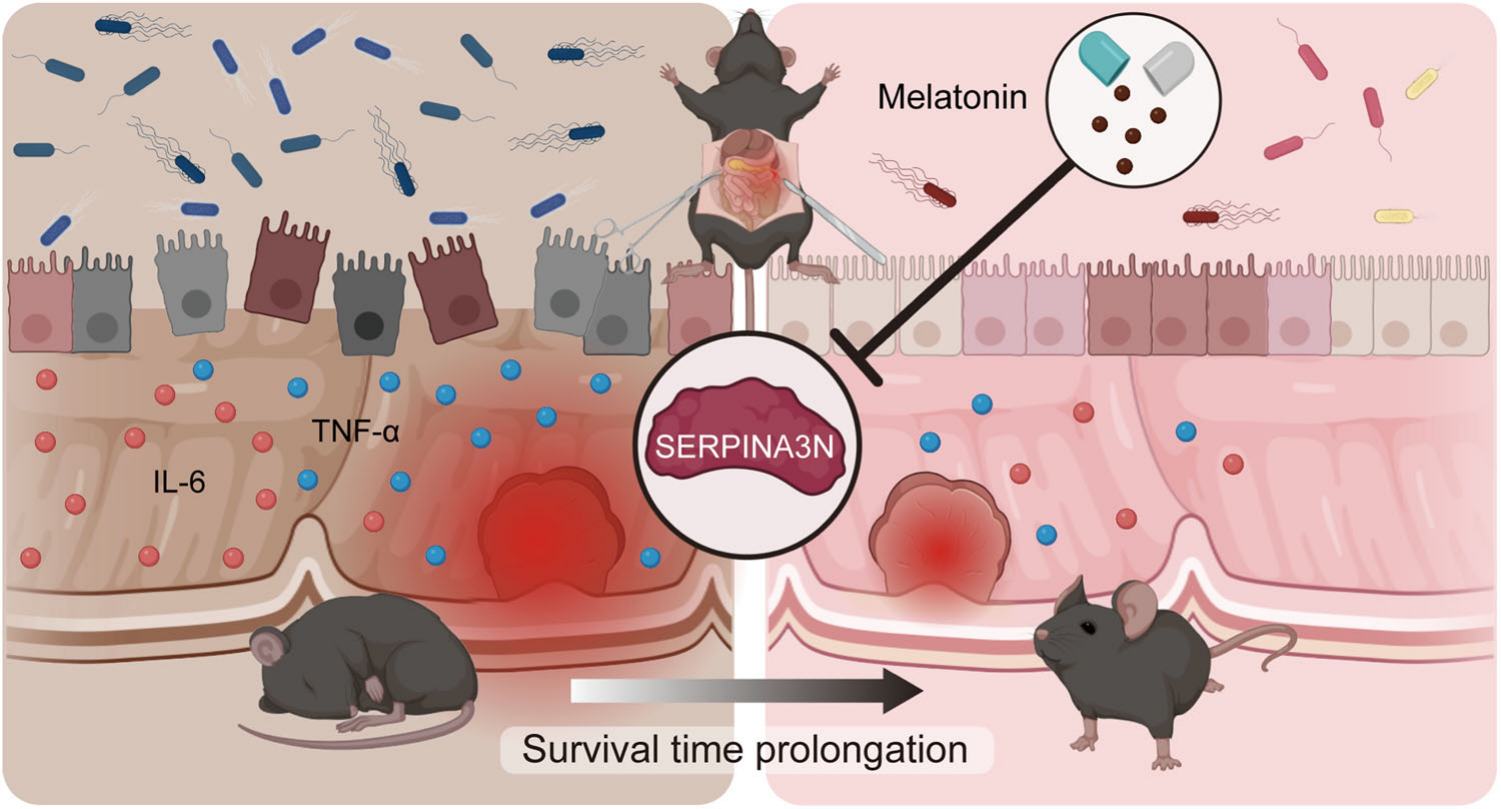
Schematic illustration of the mechanisms by which MLT administration prolongs the posttraumatic survival time of TCI mice. TCI leads to significant disruption of gut homeostasis within a short period of time, indicated by inflammatory responses, intestinal barrier hyperpermeability, and microbiota dysregulation. MLT administration can restore gut homeostasis by suppressing SERPINA3N expression in the intestinal epithelium, subsequently prolonging the survival time of TCI mice. MLT, melatonin; TCI, traumatic colon injury.

To further confirm the causality, we used JSH‐23, an inhibitor targeting the NF‐κB signaling pathway, to treat Caco‐2 cells. WB analysis showed that MLT or JSH‐23 reduced the phosphorylation of NF‐κB and SERPINA3N expression. There were no differences in the effects of MLT and JSH‐23. However, MLT could not further downregulate SERPINA3N expression on the basis of JSH‐23 treatment (Figure 5C). Likewise, MLT or JSH‐23 effectively promoted Caco‐2 cell migration. The effects of these two drugs were statistically consistent. MLT did not further facilitate the migratory capabilities of Caco‐2 cells after treatment with JSH‐23 (Figure 5D,E). Next, in vivo experiments were performed to validate these results. Mice were pretreated with 1 mg/kg JSH‐23 before 14 days of TCI modeling. A single treatment of JSH‐23 significantly extended the survival time of TCI mice, which was comparable to MLT (TCI+JSH‐23 vs. TCI+MLT: HR = 0.826, 95% CI 0.357–1.911, *p* = 0.8263). Moreover, combined use of JSH‐23 and MLT did not further promote prolongation of survival time (TCI+JSH‐23+MLT vs. TCI+JSH‐23: HR = 0.726, 95% CI 0.299–1.765, *p* = 0.4797, Figure 5F). IHC examination also showed that JSH‐23 significantly suppressed SERPINA3N expression and NF‐κB phosphorylation. No more significant effects of combinational treatment of JSH‐23 and MLT were observed (Figure 5G,H). Taken together, these data demonstrate that preventive administration of MLT can downregulate SERPINA3N expression by suppressing the NF‐κB signaling pathway, subsequently extending the posttraumatic survival time of TCI mice (Figure 6).

## DISCUSSION

Intestinal injury can be caused by multiple mechanical and nonmechanical factors. In the past few decades, the disease spectrum and therapeutic regimens of intestinal injury have significantly changed due to alterations in the social environment. Most related studies aimed to reveal the mechanisms of nonmechanical intestinal injuries, such as radiation enteritis, inflammatory bowel disease, and intestinal dysfunctions after hemorrhagic shock [21, 22, 23]. Several novel regimens have emerged due to a better understanding of intestinal injury pathogenesis. For instance, a recent clinical trial reported that dietary glutamine supplements could normalize intestinal hyperpermeability [24]. Tranexamic acid, a serine protease inhibitor, is able to attenuate gut barrier failure caused by hypoxia, ischemia–reperfusion, and hemorrhagic shock [25, 26]. Guo et al. identified the pivotal role of specific gut microbiota and metabolites in radioprotection [21]. Research on nonmechanical intestinal injuries has made tremendous progress, whereas breakthroughs in intervention strategies against traumatic intestinal injury are absent.

To the best of our knowledge, this is the first study to conduct proteomics profiling to explore critical molecules during the progression of TCI. Damage to intestinal wall integrity led to significant reprogramming of protein expression in the intestinal epithelium. Clustering analysis showed that molecules involved in inflammatory responses, metabolic disorders, tissue repair, and bacterial defense were significantly dysregulated. These findings suggested similarities in physiopathological processes between the intestines and other organs in response to traumatic injury [27, 28]. The landscape of protein reprogramming can reveal the molecular mechanisms of TCI progression and provide data support for subsequent research.

In our previous study, we generated a TCI animal model using Sprague–Dawley rats and found that disruption of gut homeostasis contributed to injury‐induced death [29]. This finding is consistent with the data based on experiments using C57BL/6 mice, further confirming the reliability of our modeling methods. A series of experiments demonstrated that TCI acutely induced gut homeostasis, indicated by inflammation, intestinal hyperpermeability, and dysbiosis. It is noteworthy that microbiota symbiosis is essential for human health, and dysregulation of microbiota has been proven to be a contributor to many diseases [30, 31]. Based on the bioinformatic analysis of metagenome data, there were no differences in alpha diversities between the sham and TCI groups, whereas beta diversity was significantly altered. These results imply that although species evenness of the gut microbiota TCI is unchanged within a short period of time after TCI modeling, the bacterial community undergoes a huge disruption and the displacement of dominant bacteria is induced.

*Shigella*, *Salmonella*, and *Escherichia* are the classical pathogenic bacteria, and their abundances are under rigid regulation in human intestines. Their rapid proliferation can induce intestinal inflammation, electrolyte disturbances, and even sepsis [32, 33, 34]. The abundances of the three pathogenic bacterial genera were significantly upregulated after damage to intestinal integrity. These above‐mentioned pathological alterations are not limited to TCI progression. Many other types of intestinal diseases, such as inflammatory bowel diseases, radiation enteritis, and metabolic disorders, also show similar pathological mechanisms [35, 36, 37]. According to the metagenome data, there may be other events of microbiota dysregulation contributing to TCI progression. For instance, the *Cronobacter* genus has been proven to be a pathogen that can cause a large number of diseases, such as meningitis, necrotizing enterocolitis, sepsis, and brain abscesses [38]. The relative abundance of *Cronobacter* was significantly upregulated in the gut of TCI mice, which may be another reason for TCI‐induced rapid death. The *Proteus* genus, another putative gastrointestinal pathogen, was found to typically proliferate in the gut [39]. The balance of *Coriobacterium*, *Lyngbya*, and many other bacteria with unknown functions was also damaged. These data suggest that the associations between TCI progression and microbiota dysregulation are complicated. More investigations should be conducted to identify important events and mechanisms of TCI and develop more effective intervention regimens.

The role of SERPINA3N in regulating intestinal disorders has not been investigated in depth and remains controversial. Zhang et al. [40] used bioinformatic analysis and a mouse model in combination to reveal its potential value in the diagnosis and therapy of ulcerative colitis (UC). SERPINA3N serves as a biomarker of active UC. Knockdown of *Serpina3n* expression attenuates inflammatory responses of intestinal epithelial cells. Nevertheless, another study used single‐cell transcriptomics to investigate the interactions of intestinal cells in an animal model of chemically induced colitis. SERPINA3N participates in the resolution of inflammation and the amelioration of colitis symptoms [41]. Our study demonstrated that SERPINA3N expression was upregulated in the intestinal mucosa after TCI modeling. Ablation of SERPINA3N effectively enhanced the migratory capability of intestinal epithelial cells in vitro and prolonged the posttraumatic survival time of TCI mice by improving gut homeostasis. The contradictions may be attributed to the characteristics of different intestinal diseases and model generation methods. SERPINA3N may act as a positive regulator of chronic inflammation. Nevertheless, the outburst of SERPINA3N expression upregulation may aggravate dysfunctions of the intestinal epithelium during the early stage of TCI progression. The delayed inhibition of SERPINA3N upregulation may prevent the progression of acute gut disorders.

Oral administration is generally recognized as the most convenient and acceptable route for medication, especially in complicated environments such as disaster‐hit areas and battlefields. Because of the critical role of SERPINA3N in the development of TCI, we searched for a highly potent inhibitor to block SERPINA3N upregulation and validate its curative effects on posttraumatic survival prolongation. Xi et al. reported that MLT can significantly inhibit SERPINA3N upregulation induced by neuroinflammation [20]. We speculated that MLT could serve as a drug for the restoration of intestinal dysregulations induced by TCI. This assumption was confirmed from our experimental data. Oral administration of MTL mitigated inflammatory responses, intestinal hyperpermeability, and rapid proliferation of pathogenic bacteria, subsequently prolonging the survival time of TCI mice. Otherwise, the safety of long‐term MLT use has already been confirmed [42]. The other properties of MLT may further improve the physical and mental health of recipients, such as sleep promotion, analgesia, and immunity enhancement [43, 44, 45]. These features confer unique advantages to MLT application for individuals under threat from TCI. MLT has excellent potential as a preventive regimen for survival extension of TCI causalities.

However, there are some limitations to the current study. First, we only proved that MLT suppresses SERPINA3N expression in the intestinal epithelium. The precise mechanisms by which MLT suppresses SERPINA3N expression still need to be investigated. Second, MLT has been reported to be a multitarget drug [46]. Our findings prove that SERPINA3N is a regulatory target in the intestinal epithelium. Whether there are other underlying targets of posttraumatic survival prolongation is unknown. Third, a mouse model was used to simulate TCI casualties. Notably, there are significant differences between animal models and humans. The value of MLT administration should be carefully evaluated by clinical trials.

## CONCLUSION

Our findings demonstrate that SERPINA3N is overexpressed in the intestinal epithelium of TCI mice, which contributes to the aggravation of inflammation, intestinal hyperpermeability, and microbiota dysbiosis. Preventive administration of MLT, a SERPINA3N inhibitor, effectively restored gut homeostasis, further prolonging the posttraumatic survival time of TCI mice. MLT medication may serve as a novel strategy of delayed treatment for high‐risk populations.

## METHODS

### Cell culture

Caco‐2, a human colon epithelial cancer cell line, was purchased from the American Type Culture Collection (ATCC, #BFN60800651). Cell lines were cultivated using Dulbecco's modified Eagle's medium (DMEM; Gibco, #C11995500BT, Thermo Fisher Scientific) with 10% fetal bovine serum (FBS; Excell Bio, #FCS500) and 1% penicillin‐streptomycin solution (100×; Gibco, #15140163). The incubator was invariably set at 37°C and 5% CO_2_.

### Pharmaceutical preparations

In the in vitro experiments, MLT solution was prepared using dimethyl sulfoxide (DMSO; MedChemExpress, #HY‐Y0320, USA) according to the indicated doses, which ensured that the volumes of DMSO that were used to treat cells did not exceed 0.5% of the volume of the cell culture medium. 100 μM JSH‐23 was also prepared with DMSO. DMSO served as the reference substance. In the in vivo experiments, the vehicle consisted of 15% Cremophor EL (MedChemExpress, #HY‐Y1890) and 85% sterile water. The oral administration doses of MLT (MedChemExpress, #HY‐B0075) and JSH‐23 (MedChemExpress, #HY‐13982) were 0.2 mg/mL and 3 mg/kg, respectively.

### Basic animal experiment

Six‐week‐old male C57BL/6 mice were purchased from SPF Biotechnology. After admission to the animal center, a 14‐day acclimatization process was followed to reduce the potential bias caused by the environment. The mice were fed under a specific pathogen‐free environment with 40%–70% relative humidity, 22 ± 2°C, and a 12 h/12 h illumination period. Mice were provided pure water and sterile standard fodder ad libitum (SPF Biotechnology). Each C57BL/6 mouse was fed in one cage.

For administration of MLT or JSH‐23, 3 mL of vehicle supplemented with drugs was added in the feeding bottle. This volume did not exceed the daily water intake of mice, which guaranteed the full administration of drugs within one day. This dose of MLT was based on the methods reported by a previous study [47]. The reference substance, namely, vehicle, was sterile water. After the mice drank the solution, they were given sterile water for the rest of the day. For prevention of MLT degradation induced by light, an aluminum foil was used to wrap the bottle surface. The euthanasia of mice was performed by gradient elevation of the CO_2_ atmosphere until confirmation of death. The samples collected from the mice were stored at −80°C if not immediately used. It is noteworthy that the sample collection was performed after 24 h of modeling. The mice used were different from the batch used for observation of survival time.

### Preparation of the TCI mouse model

Anesthesia was administered to the mice by an intraperitoneal injection of 40 mg/kg pentobarbital sodium (Hnalt Biotechnology). The mice were fixed on the operating table for small animals placed on a clean bench. The fur on the abdomen was shaved, and the skin was disinfected. The anatomical layer of the abdomen was successively incised. For simulation of TCI conditions, the incision of the colon was 3 cm from the cecum end. It was cut open semicircularly, and the wound was perpendicular to the long axis of the intestinal tract. For the mice in the sham group, incision of the colon was not performed, but the other operation processes were conducted. The wound in the abdominal wall was closed using stitches.

### Generation of *Serpina3n* KO and CKO mice

Mice with *Serpina3n* KO were established using the CRISPR‐Cas9 gene‐editing system (Cyagen Biosciences, USA). The sgRNA target sequences were introduced as follows: gRNA‐A1: GCGTTCAACTATAGTCCATT‐AGG; gRNA‐A2: CTGCAGCATCTAGGACTACT‐TGG; gRNA‐B1: AGGGGTCCAGGTTACATTAT‐TGG; gRNA‐B2: CTGGGACTGAGCTTACTTGA‐TGG. Cas9 mRNA with specific sgRNAs was injected into the fertilized eggs of C57BL/6 mice. The eggs were implanted into a pseudopregnant female surrogate mouse. We used the Cre/LoxP system (Cyagen Biosciences, USA) to cross‐breed *Serpina3n* ^flox/flox^ and Vil1‐Cre genotyped mice for multiple generations until sufficient numbers of *Serpina3n* ^flox/flox^; Vil1‐Cre mice and *Serpina3n* ^flox/flox^ mice were obtained. The Loxp primers were as follows: Loxp1: F1: 5′‐GGGGCATATGGTATGAATCTGGAA‐3′; R1: 5′‐ATATCGGATCAGTTCGTAATCCCC‐3′. Loxp2:F2:5′‐CTTTATAGCCAAGATAGCCAACCC‐3′; R2:5′‐CAAATTTCAGGCTCAGGAACATGG‐3′.

### Metagenome sequencing

For elucidation of TCI‐induced alterations of intestinal symbiosis, total genomic DNA was extracted from intestinal content samples using a PowerSoil DNA Isolation kit (MoBio Laboratories, #12888) according to the manufacturer's protocol. The quality and quantity of the extracted DNA were examined using a Qubit dsDNA HS Assay Kit on a Qubit 3.0 Fluorometer (Life Technologies, #Q33216). A VAHTS Universal Plus DNA Library Prep Kit for Illumina (Vazyme Biotech, #ND617‐01) was used to construct paired‐end libraries. The library was sequenced on an Illumina NovaSeq. 6000 platform (Biomarker Technology) using the 150‐bp paired‐end sequencing mode. After trimming the adaptors and filtering the low‐quality reads, the clean sequence data were further analyzed using the software R package.

### Proteomics profiling

Label‐free quantitative proteomics was used to investigate the effects of TCI on protein expression in the intestinal epithelium. The intestinal mucosae of sham and TCI mice were collected and lysed with grinders and lysis buffer. The tissue debris was removed by centrifugation. The protein concentrations of the samples were determined using a Pierce ECL Plus Kit (Thermo Scientific, #32132). An EASY‐nLC 1000 System (Thermo Scientific) and an Orbitrap Fusion Mass Spectrometer (Thermo Scientific, #IQLAAEGAAPFADBMBHQ) were used in combination to record the protein spectra of the samples. Further data analysis was performed using MaxQuant software.

### Establishment of cell lines with stable interference of SERPINA3N expression

The plasmids carrying shRNA targeting *Serpina3n* or *Serpina3n* overexpression sequences were designed and synthesized by JTS Scientific. A lentiviral packaging kit (Yeasen, #41102ES40 was used to prepare lentiviruses carrying stable interference sequences according to the protocol. Caco‐2 cells were subsequently cultivated with lentivirus for cell infection. Puromycin (5 μg/mL) was used to chemically select cells that were successfully infected.

### qRT‐PCR

To determine the relative abundances of specific pathogenic bacteria, the colon contents were harvested from mice and total bacterial DNA was extracted using a QIAamp FAST DNA Stool Mini Kit (Qiagen; Dusseldorf, #51604). The quality of the extract was measured by Microdrop (BIO‐DL). The DNA samples were amplified using SYBR Premix Ex Taq II (TaKaRa, #RR820A). The 2^−ΔΔCt^ method was performed to calculate the relative abundances of specific genera. The primer sequences for qRT‐PCR are listed in Supporting Information: Table S1.

### Western blot analysis (WB) analysis

The collected samples were washed with cold phosphate‐buffered saline and lysed using radioimmunoprecipitation assay buffer (Biorigin, #BN25012) with protease inhibitor cocktail (Biorigin, #BN25002). The sample debris was removed by high‐speed centrifugation. Next, the proteins were subjected to gel electrophoresis according to the protein sizes and transferred onto polyvinylidene difluoride membranes (Millipore, #V515818, Merk). Then, 5% defatted milk was prepared to block the membranes for 1 h at room temperature. The membranes were then incubated with primary antibodies at 4°C overnight and then with secondary antibodies at room temperature for 2 h. The antibodies and the corresponding dilutions are as follows: anti‐SERPINA3N antibody (Bioss, #bs‐0094R, 1:1000), anti‐NF‐κB antibody (Abcam, #ab32536, 1:1000), anti‐p‐NF‐κB antibody (Abcam, #ab86299, 1:1000), anti‐β‐actin antibody (Abcam, #ab32536, 1:1000), and anti‐rabbit IgG (Abcam, #ab6721, 1:3000). The blots were imaged using a Pierce ECL Plus Kit.

### Immunohistochemistry (IHC) examination

An IHC assay was conducted to determine ZO‐1 and occludin expression in situ in the intestinal mucosa. Briefly, the tissues were fixed using 4% paraformaldehyde (Servicebio, #G1101, Beijing, China) and embedded in paraffin. A slicer was used to prepare sections that were mounted on sample slides. After deparaffinization, the slides were stained using specific primary and secondary antibodies. The used antibodies and their corresponding dilutions are as follows: anti‐ZO‐1 antibody (Abcam, #ab221547, 1:200), anti‐Occludin antibody (Abcam, #ab216327, 1:200), anti‐SERPINA3N antibody (Bioss, #bs‐0094R, 1:200), anti‐NF‐κB antibody (Abcam, #ab32536, 1:200), anti‐p‐NF‐κB antibody (Abcam, #ab86299, 1:200), and anti‐rabbit IgG (Abcam, #ab6721, 1:1000). The IHC score evaluation was also conducted as described in our previous study [29].

### Transwell assay

To analyze the effects of SERPINA3N and MLT on the migratory capabilities of intestinal epithelial cells, 5 × 10^4^ treated Caco‐2 cells were harvested and cultivated in FBS‐free DMEM. Then, they were seeded in the upper chamber of transwells (Corning, #3460) and the bottom chamber was filled with 600 μL of DMEM with 20% FBS. 24‐well plates loaded with transwells were placed in the incubator. After 24 h, the transwells were fixed with 4% paraformaldehyde and cells that migrated to the lower chamber were stained with 0.1% crystal violet (Biorigin, #BN24042). The cells were counted under an optical microscope (Leica).

### Enzyme‐linked immunosorbent assay (ELISA)

ELISA was conducted to measure the levels of indicators of inflammation and intestinal permeability. Peripheral blood was harvested and centrifuged at 1800*g* for 10 min to prepare plasma. ELISA kits for the detection of endotoxin (Enzyme‐linked Biotechnology, #ml‐E6008, Shanghai, China), D‐lactate (Nanjing Jiancheng, #A019), DAO (Enzyme‐linked Biotechnology, #ml‐E4175), IL‐6 (Abbkine, #KTE7009), TNF‐α (Abbkine, #KTE7015), and CRP (Multi Sciences, #EK294/2) were used according to the manufacturers' protocols. The absorbances of the samples were determined using a microplate reader (BioTek). The relative level of each group was calibrated based on the control group.

### Statistical analysis

The statistical analysis, survival comparison, and data presentation were performed using Prism 8.0 and SPSS 25.0. The Shapiro–Wilk test was conducted to confirm data normality. Data are presented as the mean ± standard deviation (SD). Student's *t*‐test was used to compare differences between two groups of data containing one variable with normal distributions. One‐way analysis of variance (ANOVA) was used to compare differences between three or more groups of data containing one variable with normal distributions. For the data containing two variables, two‐way ANOVA was used to compare statistical differences. The Kruskal–Wallis test was used to compare the differences between groups without normal distributions. Comparisons of survival time were conducted using the log‐rank (Mantel‐Cox) test. Two‐sided *p* < 0.05 was considered significant.

## AUTHOR CONTRIBUTIONS

Bo Cao, Jing‐Wang Gao, Qing‐Peng Zhang, and Xing‐Ming Xu performed the in vivo experiments. Bo Cao, Jing‐Wang Gao, Rui‐Yang Zhao, and Hang‐Hang Li conducted basic and in vitro experiments. Bo Cao, Qing‐Peng Zhang, and Xing‐Ming Xu wrote the manuscript and interpreted the data. Bo Wei designed the project, supervised the experimental processes, and revised the manuscript. All authors read and approved the final version of the manuscript. Co‐first authorship was decided based on crucial contributions to experiments and leadership.

## CONFLICT OF INTEREST STATEMENT

The authors declare no conflict of interest.

## ETHICS STATEMENT

The animal studies were approved by the Ethics Committee of Animal Centre of Chinese PLA General Hospital (2020‐X6‐117).

## Supporting information

Supporting information.

Supporting information.

Supporting information.

Supporting information.

Supporting information.

Supporting information.

Supporting information.

Supporting information.

Supporting information.

Supporting information.

## Data Availability

The data sets used and analyzed during the current study are available from the corresponding author on reasonable request. The raw data reported in this study are available in Science Data Bank (https://www.scidb.cn/en/s/fuiAJn). Supplementary materials (figures, tables, scripts, graphical abstract, slides, videos, Chinese translated version, and update materials) can be found in the online DOI or iMeta Science http://www.imeta.science/.
