# Supplementary material for: Melatonin supplementation protects against traumatic colon injury by regulating SERPINA3N protein expression
Source: Imeta. 2023 Oct 24;2(4):e141. doi: 10.1002/imt2.141 (PMC10989984; doi:10.1002/imt2.141)
Supplement: Supplementary file 2 — Supporting information. [file IMT2-2-e141-s004.docx]

**Supporting information to:**

Melatonin supplementation protects against traumatic colon injury through regulating SERPINA3N protein expression

**Running title**

The role of melatonin in alleviating traumatic colon injury

**Authors**

Bo Cao^1,†^, Jing-Wang Gao^1,2,†^, Qing-Peng Zhang^1,†^ Xing-Ming Xu^1,^, Rui-Yang Zhao^1,2^, Hang-Hang Li^1,2^, Bo Wei^1,*^

^1^ Department of General Surgery, First Medical Center, Chinese PLA General Hospital, Beijing 100853, China.

^2^ Medical School of Chinese PLA, Beijing 100853, China.

^†^ These authors contributed equally to this work.

***Corresponding author**

Dr. Bo Wei MD, PhD

Deputy Director of Department of General Surgery

Institute of General Surgery/Department of General Surgery

First Medical Center, Chinese PLA General Hospital

Beijing 100853, China.

Tel: +86-10-66938071

Fax: +86-10-68181689

E-mail: [weibo@301hospital.com.cn](mailto:weibo@301hospital.com.cn)

**Figure S1.** qRT-PCR analysis to determine the relative abundances of *Cronobacter* and *Proteus* in intestinal contents of sham and TCI mice (n = 15 per group). *****p* < 0.0001.

**Figure S2.** SERPINA3N inhibits migration capabilities of intestinal epithelial cells. (A) The WB analysis to detect SERPINA3N expression in Caco-2 cells with SERPINA3N overexpression and knockdown and the control cells. (B) The transwell assay to determine cell migration capabilities of cells as in (A). Scale bar: 100 μm. (C) The histogram to show the relative cell migration rate of cells as in (B). ***p* < 0.01, ****p* < 0.001.

**Figure S3.** The IHC examination to detect the SERPINA3N expression in the intestinal epithelium of WT and *Serpina3n* KO mice. Scale bar: 100 μm (n = 15 per group).

**Figure S4.** Conditional knockout of *Serpina3n* prolongs the survival time of TCI mice. (A) WB analysis to determine the absence of SERPINA3N expression in the intestinal epithelium of *Serpina3n* CKO mice. (B) The survival curve to compare survival time of *Serpina3n* CKO, *Serpina3n* KO and control mice after TCI modelling (n = 15 per group).

**Figure S5.** Knockout of SERPINA3N protein prolongs and gut homeostasis of TCI mice. (A) ELISA assay to detect the concentrations of CRP in plasma of WT or *Serpina3n* KO mice that received sham or TCI operation (n = 15, 15, 11 and 13, respectively). *****p* < 0.0001, n.s not significant. (B) ELISA assay to detect the concentrations of DAO, D-lactate and endotoxin in plasma of mice as in (A). (C) qRT-PCR analysis to determine the relative abundances of *Cronobacter* and *Proteus* in intestinal contents of mice as in (A). ***p* < 0.01, ****p* < 0.001, *****p* < 0.0001, n.s not significant.

**Figure S6.** The effects of MLT on SERPINA3N expression of gut epithelium *in vitro* and *in vivo*. (A) WB analysis to determine the SERPINA3N expression in Caco-2 cells treated with 0.01, 0.1 and 1 μM MLT for 24 h. (B) The transwell assay to determine cell migration capabilities of cells as in (A). The histogram is displayed on the right. Scale bar: 100 μm. (C) The IHC examination to detect the SERPINA3N expression in the intestinal epithelium of mice in the sham or TCI groups that were administered with vehicle or MLT as indicated (n = 15 per group). The histogram is displayed on the right of pictures. Scale bar: 100 μm. **p* < 0.05, ***p* < 0.01, ****p* < 0.001.

**Figure S7.** SERPINA3N is a downstream target of MLT to prolong survival time of TCI mice. (A) WB analysis to determine the SERPINA3N expression in Caco-2 cells with or without SERPINA3N overexpression treated with vehicle or MLT. (B) The transwell assay to determine cell migration capabilities of cells as in (A). Scale bar: 100 μm. (C) The histogram to show cell migration rates as in (B). (D) The survival curve to compare survival time of WT or *Serpina3n* KO mice that received TCI operation treated with vehicle or MTL. ***p* < 0.01, ****p* < 0.001.

**Figure S8.** Administration of MLT extends the survival time and gut homeostasis of TCI mice via downregulation of SERPINA3N expression. (A) ELISA assay to detect the concentrations of CRP in plasma of mice in the sham or TCI group that were administered with vehicle or MLT (n = 15, 15, 10 and 13, respectively). (B) ELISA assay to detect the concentrations of DAO, D-lactate and endotoxin in plasma of mice as in (A). (C) qRT-PCR analysis to determine the relative abundances of *Cronobacter* and *Proteus* in intestinal contents of mice as in (A). ****p* < 0.001, *****p* < 0.0001, n.s not significant.
