# Supplementary figures and images for: Melatonin supplementation protects against traumatic colon injury by regulating SERPINA3N protein expression
Source: Imeta. 2023 Oct 24;2(4):e141. doi: 10.1002/imt2.141 (PMC10989984; doi:10.1002/imt2.141)

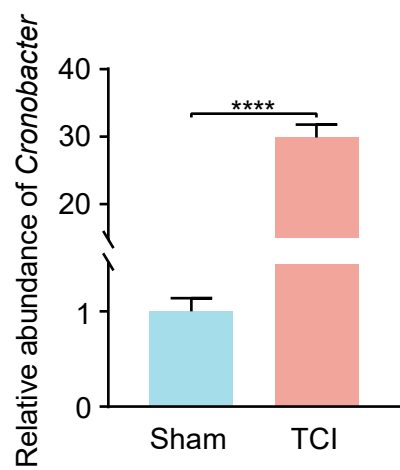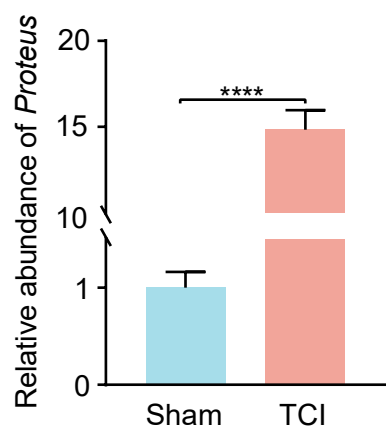

Supplement: Supplementary file 3 — Supporting information. [file IMT2-2-e141-s003.pdf]

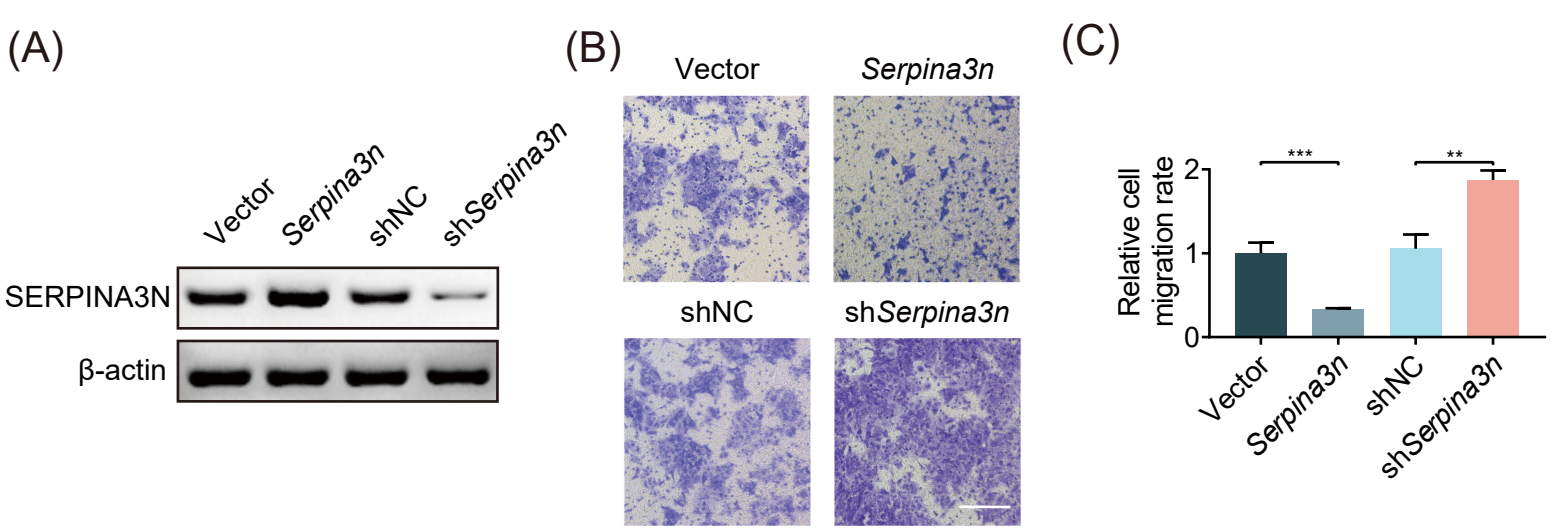

Supplement: Supplementary file 4 — Supporting information. [file IMT2-2-e141-s002.pdf]

(A)

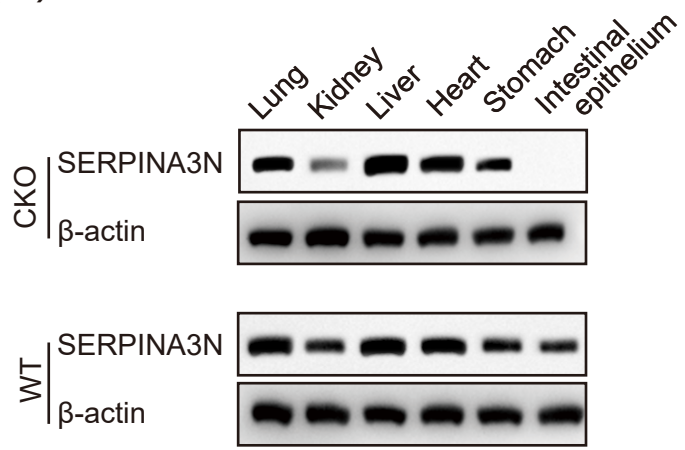

(B)

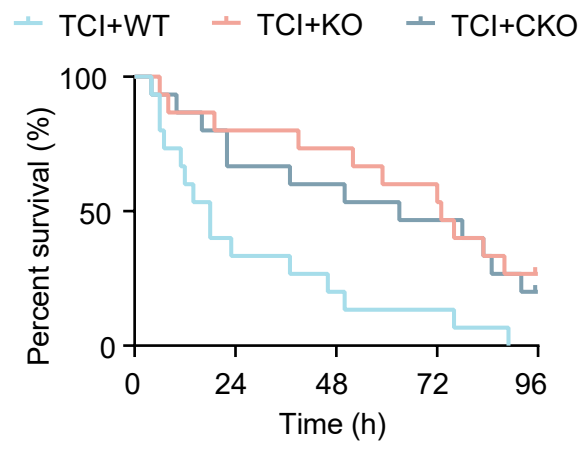

Supplement: Supplementary file 6 — Supporting information. [file IMT2-2-e141-s010.pdf]

(A)

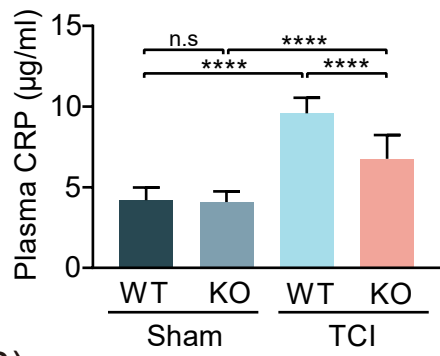

(C)

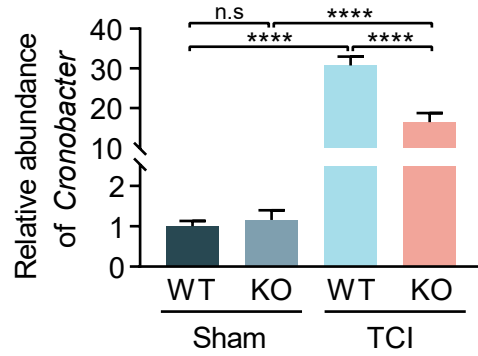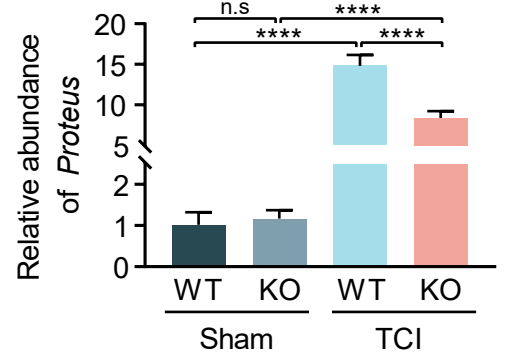

(B)

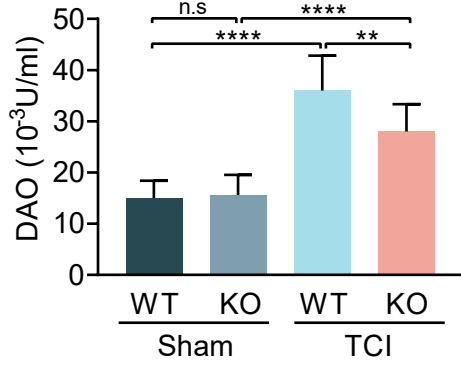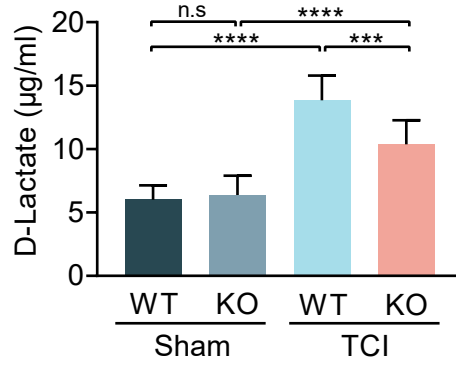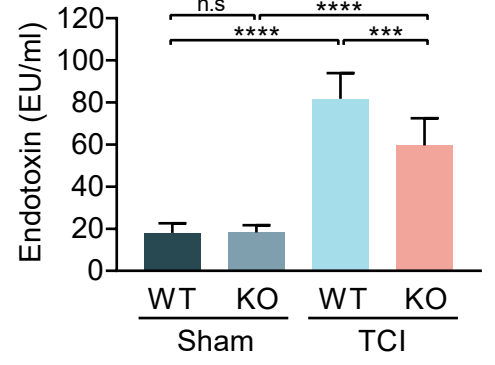

Supplement: Supplementary file 7 — Supporting information. [file IMT2-2-e141-s005.pdf]

(A)

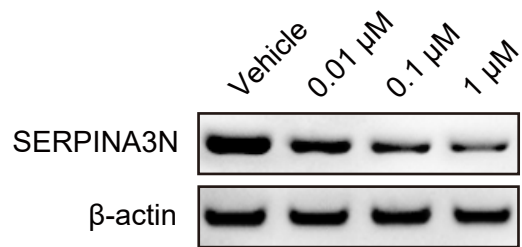

(B)

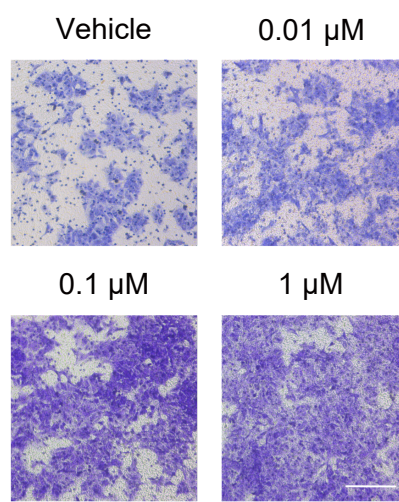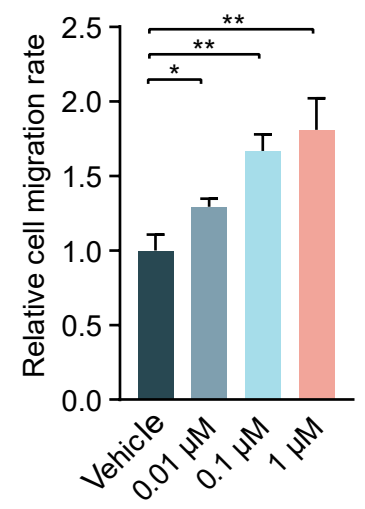

(C)

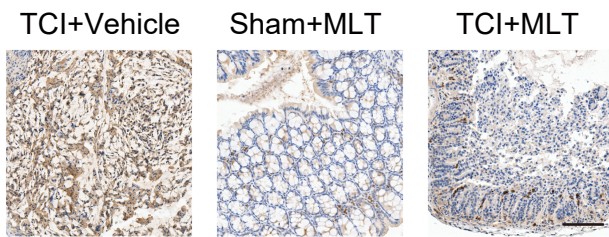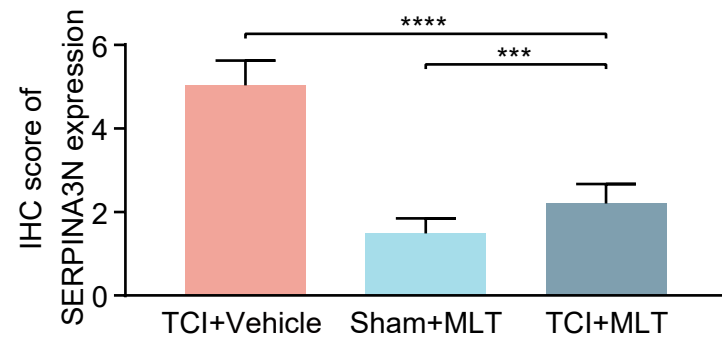

Supplement: Supplementary file 8 — Supporting information. [file IMT2-2-e141-s006.pdf]

(A)

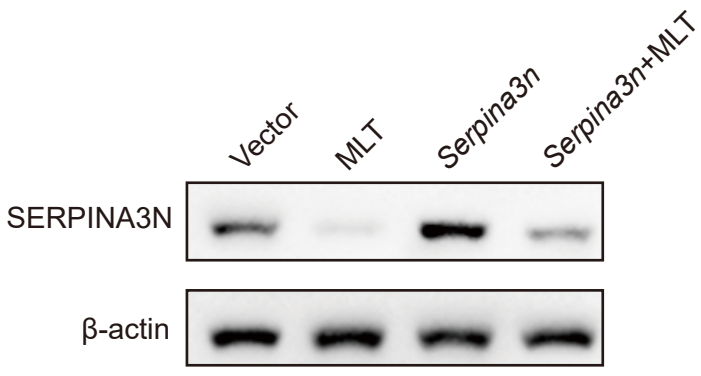

(B)

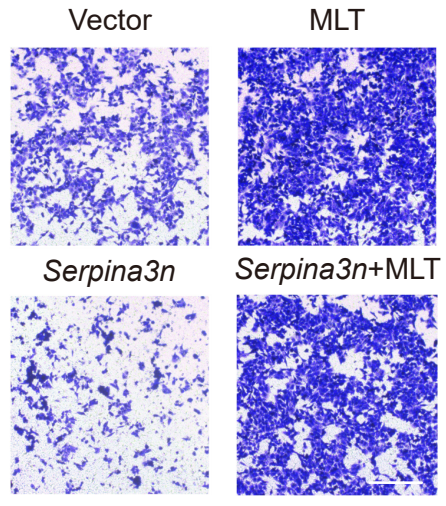

(C)

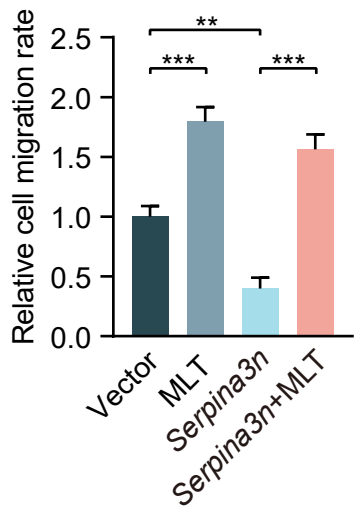

(D)

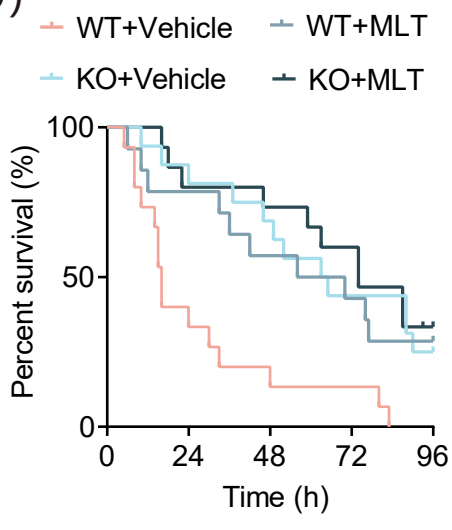

Supplement: Supplementary file 9 — Supporting information. [file IMT2-2-e141-s001.pdf]

(A)

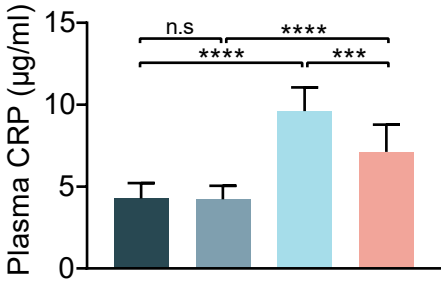

(C)

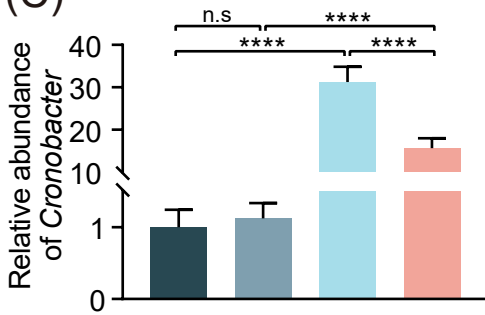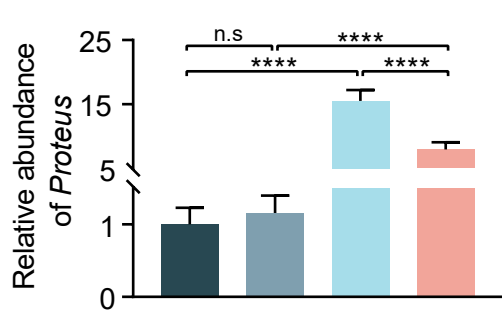

(B)

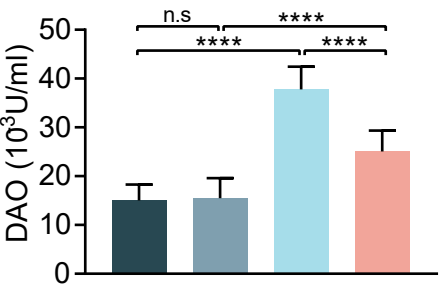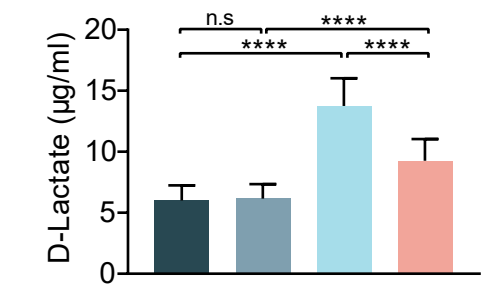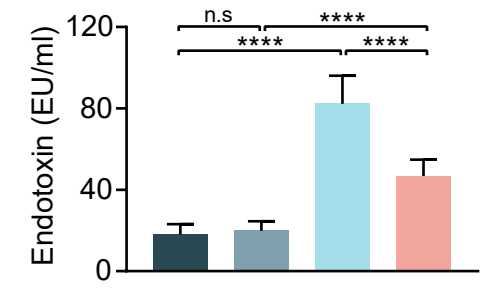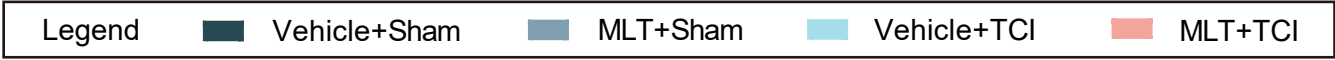

Supplement: Supplementary file 10 — Supporting information. [file IMT2-2-e141-s009.pdf]
