# Supplementary material for: Melatonin supplementation protects against traumatic colon injury by regulating SERPINA3N protein expression
Source: Imeta. 2023 Oct 24;2(4):e141. doi: 10.1002/imt2.141 (PMC10989984; doi:10.1002/imt2.141)
Supplement: Supplementary file 5 — Supporting information. [file IMT2-2-e141-s008.pdf]

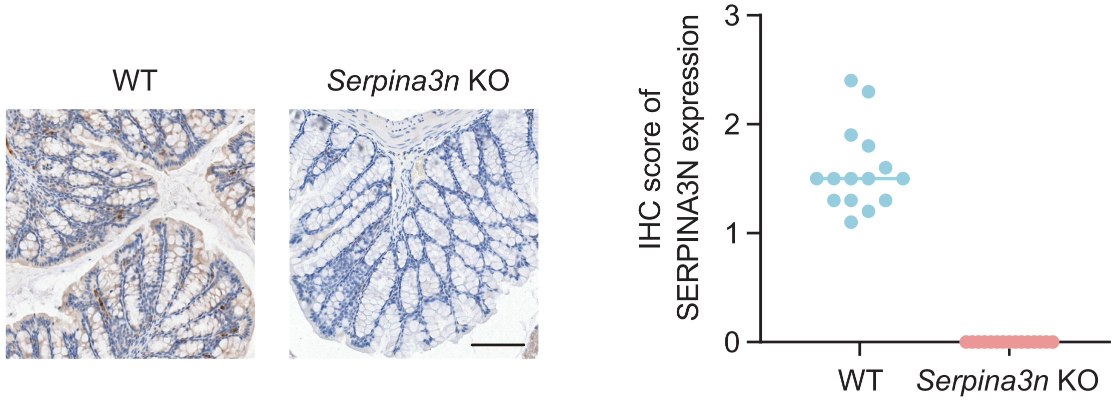

WT
Serpina3n KO
IHC score of SERPINA3N expression
3
2
1
0
WT
Serpina3n KO
